# Collective behavior of higher-order globally coupled oscillatory networks in response to positive and negative couplings

**DOI:** 10.3389/fnetp.2025.1582297

**Published:** 2025-07-22

**Authors:** Lixin Yang, Mengjiao Li, Jun Jiang

**Affiliations:** ^1^ School of Mathematics and Data Science, Shaanxi University of Science and Technology, Xi’an, China; ^2^ State Key Laboratory for Strength and Vibration, Xi’an Jiaotong University, Xi’an, China

**Keywords:** complex network, higher-order interactions, network physiology, synchronous behavior, coupling patterns

## Abstract

Collective behavior is among the most fascinating complex dynamics in coupled networks with applications in various fields. Recent works have shown that higher-order interactions widely exist in complex systems. Both positive couplings among nodes, as the majority of studies have assumed, and negative couplings are very common in real-world systems, like physiological networks. Positive coupling (excitatory coupling) promotes synchronization and drives excitatory synaptic transmission between neurons. Meanwhile, negative coupling (inhibitory coupling) inhibits synchronization and sustains inhibitory synaptic transmission between neurons. Since high-order coupling patterns and different coupling patterns strongly affect the synchronous performance of complex systems, this article develops a globally coupled higher-order oscillatory system model that incorporates both positive and negative couplings. It is shown that, in the case of positive couplings, a second-order interaction has a negligible impact on the synchronization capability of a network within a certain range. In contrast, a higher-order network with purely negative couplings exhibits asynchronous states for any values of the second-order interactions. However, the synchronous region gradually shrinks with the increase of the negative coupling in the case of mixed couplings. This indicates a prominent role of coupling patterns on the onset of globally higher-order network synchronization.

## 1 Introduction

Both power networks and neuronal networks featuring synaptic plasticity describe real-world complex systems of critical importance in modern times (Parshani and Stanley, 2011). The majority of our infrastructure and activities crucially depend on a reliable supply of electric power. Hence, various real-world networks have been successfully modeled as coupled dynamical systems with many interacting units. In general, complex dynamic networks can be regarded as ensembles of nodes with various dynamics connected by links, where the connections denote pairwise interactions (Grzybowski et al., 2016; Gomes-Gardenes et al., 2007; Fan et al., 2018). The majority of previous studies have focused on analyzing networks with pairwise interactions, thereby neglecting the higher-order interactions that can exist between these networks. Indeed, pairwise interactions fall short when describing any realistic process responses and fail to describe practical situations.

Researchers have developed a general method for analyzing the attribution of symmetry breaking. Their findings give the first demonstration of symmetry breaking in power grids (Nishikawa and Motter, 2021). Instead, higher-order interactions among nodes have been discussed in the context of the characteristics of complex system structures and dynamics. Specifically, higher-order interactions (Battiston et al., 2020; Boccaletti et al., 2023) contain hypergraphs and simplicial complexes (Zhang et al, 2023; Xu et al., 2021). Recent studies have revealed that the presence of higher-order interactions plays a significant role in the dynamics of networked systems (Dai et al., 2021; Bairey et al., 2016; Zhang et al., 2023; Skardal and Arenas, 2020a). The higher-order interaction plays a critical role in shaping complex systems and their collective behaviors. It is well known that the performance of the dynamic behavior of networks has been significantly influenced by coupling patterns (Gambuzza et al., 2021; Tlaie et al., 2019). In recent years, some efforts have been devoted to studying the dynamic behaviors of complex networks with higher-order structures because of this special characteristic being more accurate while analyzing dynamic behaviors for real-world complex networks (Estrada and Ross, 2018; Battiston et al., 2020; Grilli et al., 2017; Benson et al., 2016; Witthaut et al., 2016).

Researchers have also investigated the optimization of collective behavior in networks with higher-order interactions encoded in clique complexes. This work demonstrates that strengthening higher-order couplings enhances collective behavior and broadens the range of possible dynamics, with an ideal balance between pairwise and higher-order interactions yielding the strongest collective behavior (Skardal et al., 2021). In particular, synchronous behavior is a phenomenon appearing in many real complex systems. The majority of systems exhibit striking similarities in their behavior when passing from a disordered to an ordered state. Many researchers have investigated the relationship between coupling patterns and synchronized states. Self-organized synchronization in physiological networks can potentially lead to epilepsy, cardiac arrhythmias, or immune dysregulation. Gaining a deeper understanding of these synchronization mechanisms offers targets for disease intervention strategies. Kuramoto oscillators describe phase synchronization and are applicable to the study of the rhythmic coordination within neuronal populations. Authors investigated the dynamics of Kuramoto oscillators with higher-order structures and found the emergence of explosive synchronization (Rodrigues et al., 2016). Fatemeh et al. (2022) studied the effects of pairwise and three-body interactions on the emergence of synchronization in Hindmarsh–Rose neurons. Their results indicated that the overall synchronization cost is reduced due to the introduction of three-body interactions. Additionally, Skardal and Arenas studied the dynamics of phase oscillators with higher-order interactions. They found that the higher-order structure may cause self-organized features and achieve synchronization of the overall system (Skardal and Arenas, 2020a).

Power systems are one of the most critical infrastructures in the real world. These systems can be modeled as complex networks that contain generations, electricity consumers, and transmission lines (Schäfer et al., 2018; Yang et al., 2023; Mandal and Banerjee, 2016; Witthaut et al., 2022). All of these components are linked by various interactions. The majority of existing results focused only on power systems with single connectivity, which does not take into account the effects of higher-order structures in the real power system. In fact, there exist pairwise and higher-order interactions among elements simultaneously. However, the crucial role of higher-order structures is still unclear in the coupled oscillatory power systems. Hence, it is necessary to investigate the relationship between higher-order structures and complex dynamics in power systems.

The second-order swing equation provides a standard dynamical model of the power system (Filatrella et al., 2008). This model has stimulated further studies in the field of power systems (Berner et al., 2021; Frasca and Gambuzza, 2021; Nauck et al., 2022). For instance, Berner, Yanchuk, and Schöll offered profound insights into the fundamental relationship between power grid networks and neuronal networks. Their findings proved that phase oscillator models with inertia were applicable to more general categories of power grid models. Moreover, they uncovered a plethora of multicluster states for phase oscillators with inertia (Berner et al., 2021). León et al. (2024) studied a globally coupled identical oscillator model, revealing the important role of higher-order interactions in synchronization transitions and multistability by introducing a three-body interaction with a phase lag. Particularly, when the coupling strength or phase lag varies, the system exhibits different synchronized, incoherent, and two-cluster states. Higher-order structures are ubiquitous in such networks and profoundly influence their dynamic behaviors.

Synchronization is among the most important collective behaviors in coupled oscillatory systems. In 1977, Hermann Haken investigated how biological networks spontaneously organize into ordered structures through the collective interactions of subsystems. His work demonstrated that even disordered systems can transition to a state of coherence via order parameters, which dominate the macroscopic behavior of the networks. This framework has become foundational for modeling networks in neuroscience and power systems (Haken, 1977). Most importantly, this behavior plays a vital role in the reliable operation process of power systems (Rohden et al., 2012; Menck et al., 2014; Schäfer et al., 2015). The robust operation of a power system relates to the synchronization of all elements from the perspective of a complex network. Therefore, due to the importance of synchronization emergence in real-world complex networks, many researchers have been motivated to study the mathematical aspects of synchronization and its influential factors. For example, Wilson et al. (2018) discussed the synchronous behavior of coupled oscillators with weak and strong coupling. They derived the expression of upper bounds on the critical coupling strength of networks under different perturbations and predicted synchronization using graph theoretical techniques. Taher H. et al. investigate the synchronization and stability of power grids using the Kuramoto model with inertia, focusing on time-delayed feedback control strategies to achieve synchronization and Lyapunov stability across different network configurations and models (Taher et al., 2019). Costa et al. (2024) combined external periodic forcing and higher-order interactions in the Kuramoto model, revealing a rich bifurcation scenario that produced 11 distinct asymptotic states and demonstrated the competition between forced and spontaneous synchronization. The dynamics of coupled oscillator networks with higher-order interactions and their ability to store information have been studied in recent works. For instance, Skardal and Arenas (2020b) propose a stability criterion to identify stable states in such systems while also exploring how these systems switch between stable states under random perturbations. In an extension of the Kuramoto model, Xu and Skardal (2021) explore three-way simplicial interactions, revealing novel dynamical properties such as clustering, multistability, and abrupt desynchronization transitions, while providing a rigorous spectral analysis of the stability of multicluster states in the thermodynamic limit. Additionally, researchers have demonstrated that phase frustration in networks of phase-frustrated coupled oscillators with higher-order interactions can unexpectedly promote explosive synchronization, a result explained through a low-dimensional model and bifurcation analysis (Dutta et al., 2023).

Later, researchers investigated synchronization of Kuramoto oscillators for power grids with general connectivity and damping (Choi and Li, 2019). Results are given as an estimate for a synchronous basin in a power system with general damping. Tang et al. investigated synchronous performance in a multilayer network. They proposed an approximation method of enhancing the predictive power for stable synchronization in multilayer networks (Tang et al., 2022). In 2023, Chen et al. revealed that the power system has higher-order connectivity features and studied the influence of topology structure on stability and construction cost (Liu et al., 2023). As power grids become more complex due to renewable energy integration and large-scale expansions, maintaining synchronization and preventing cascading failures are major challenges. Totz et al. (2020) introduced a two-layer control scheme for power grids, where the first layer represents the grid itself and the second layer manages frequency synchronization. Their study shows that a control strategy minimizing frequency differences between nodes can effectively handle various perturbations. Olmi et al. (2024) expanded on this by proposing a multilayer network control system to address node failures and cascading line failures. Their study of an Italian high-voltage grid demonstrates that distributed proportional and integral control laws can stabilize the grid even under extreme conditions. Schäfer et al. (2022) explored Braess’ paradox in power grids, showing that adding transmission lines can sometimes reduce system performance and cause blackouts. Their work emphasizes the importance of considering network topology when expanding grid capacity.

Despite many theoretical advances, little attention has so far been given to the performance of higher-order power systems with multiple coupling patterns. In reality, coupled oscillators are subjected to a mixture of both positive and negative couplings. From an energy perspective, multiple couplings are more beneficial for power transmission. Therefore, it is of great interest to study the collective dynamics of real-world networks with multiple coupling types, as this interaction substantially impacts the system’s critical phenomena.

In light of these concerns, this article addresses the scenario in which positive and negative interactions coexist in a high-order power system. Moreover, we further analyze the complex behaviors of the proposed model. Specifically, the synchronous solution is derived through theoretical analysis, and the influence of higher-order structures on synchronous stability is discussed.

The remainder of this article is organized as follows. First, we present a globally coupled higher-order oscillatory power system model with mixed coupling types in Section II. Then, in Section III, stability analysis for the condition of the synchronous solutions of a globally coupled higher-order oscillatory network system is investigated. In Section IV, we test our theory on higher-order coupled networks and perform numerical simulations to analyze the influence of coupling patterns. Section V concludes the article.

## 2 Mathematical model

**FIGURE 1:**
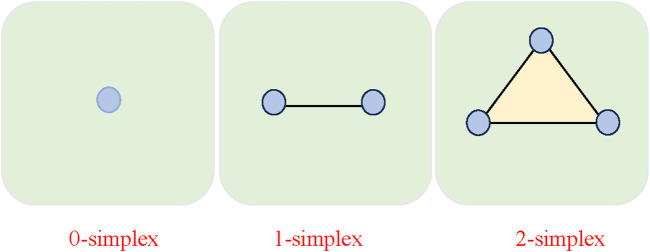
The building blocks of the higher-order interactions consist of nodes (0-simplices), edges (1-simplices), and triangles (2-simplices).

To study the dynamics of a power system with coupled oscillators, we assume that the oscillators evolve through first- and second-order interactions. Then, the general mathematical model can be described by the following Equation 1:

$${\dot{X}}_{i}=f\left(X_{i}\right)+K_{1}\sum_{j=1}^{N}a_{ij}^{\left(1\right)}h_{1}\left(X_{i},X_{j}\right)+K_{2}\sum_{j=1}^{N}\sum_{j=1}^{N}a_{ijk}^{\left(2\right)}h_{2}\left(X_{i},X_{j},X_{k}\right)$$ (1)

where $X_{i}$ denotes the state variable of the network, $f\left(X_{i}\right)$ describes the dynamic behavior of the nodes, and $K_{1},K_{2}$ are the coupling strengths associated with the first- and second-order interactions, respectively. The coupling functions are given by $h_{1}\left(X_{i},X_{j}\right),h_{2}\left(X_{i},X_{j},X_{k}\right)$ . Moreover, the adjacency matrix of the first-order coupling is denoted by $A^{\left(1\right)}=\left[a_{ij}^{\left(1\right)}\right]$ . We allow the elements of the matrix to take on three values. There, a three-oscillator interaction is represented by a 2-simplex, and any $\left(d+1\right)$ -oscillator interaction is represented by a d -simplex (also called a simplex of order d ), as illustrated in Figure 1. Specifically, we randomly select the value of $a_{ij}^{\left(1\right)}$ with

$$a_{ij}^{\left(1\right)}=\left\{\begin{array}{l} -1\begin{array}{cc} , & \text{with}\begin{array}{cc} & \text{probability}\begin{array}{cc} & \eta , \end{array} \end{array} \end{array}\\ 1,\begin{array}{cc} & \begin{array}{cc} \text{with} & \text{probability}\begin{array}{cc} & 1-\eta , \end{array} \end{array} \end{array} \end{array}\right.$$

**FIGURE 2:**
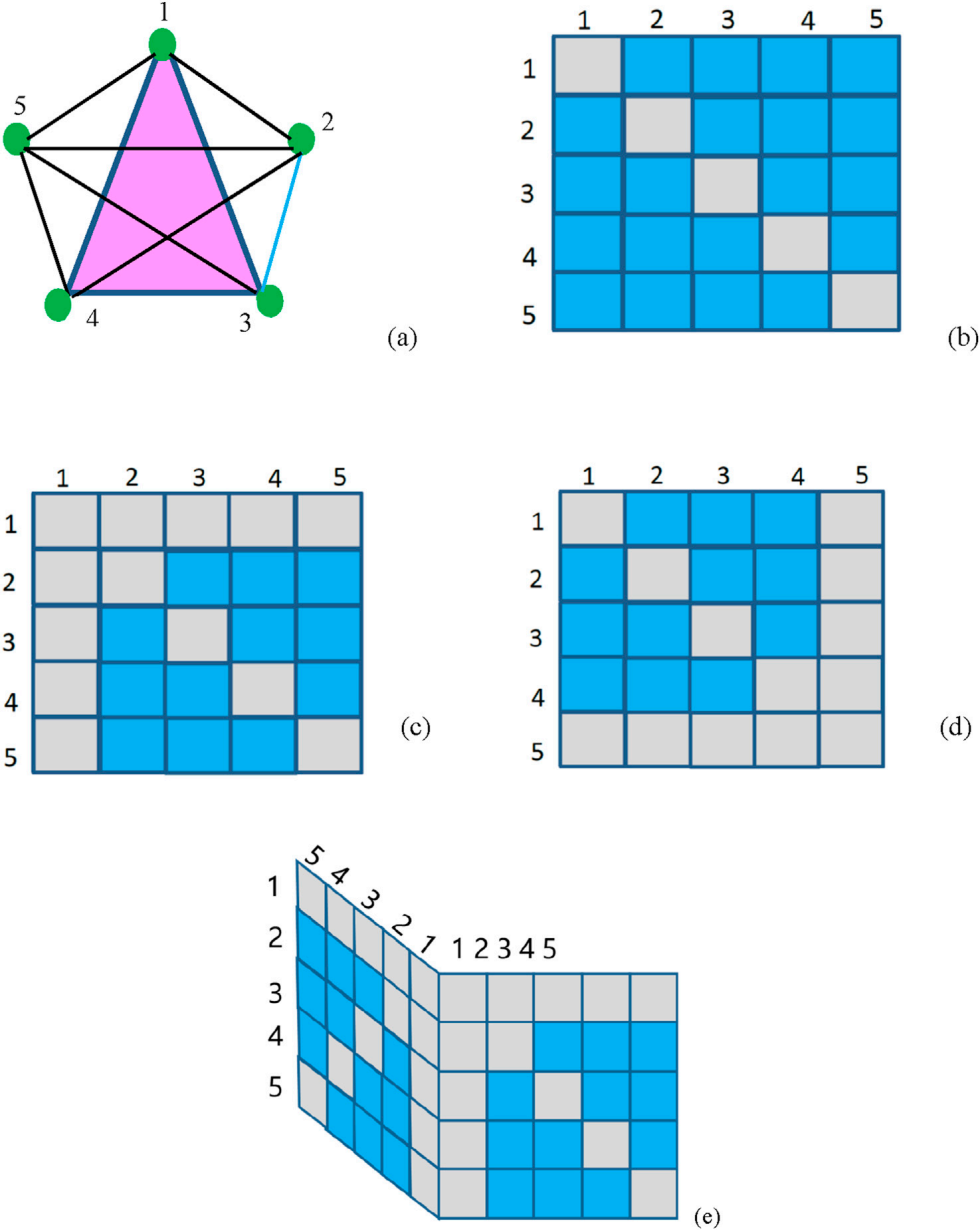
**(a)** Schematic illustration of a five-node network with global coupling. A first-order (blue) and a second-order (triangle) of the network are described by blue and pink colors as an example. **(b)** The corresponding adjacency matrix $A^{\left(1\right)}$ . The elements depicted in blue represent a link between nodes *i* and *j*. **(c)** This panel represents the second-order adjacency tensor $A^{\left(2\right)}\left(1,:,:\right)$ , which is a three-dimensional tensor that encodes interactions between triplets of nodes, specifically forming triangles in the network. Each entry in this tensor corresponds to a specific triangle (set of three interconnected nodes). The blue blocks in the matrix indicate the presence of these triangles. For example, if nodes $\left(1,:,:\right)$ form a triangle, the corresponding element in the tensor is blue. The presence of blue elements in this matrix represents the second-order interactions, meaning that these interactions occur between three nodes at once, rather than only between pairs of nodes (as in first-order interactions). **(d)** The corresponding adjacency tensor $A^{\left(2\right)}\left(5,:,:\right)$ . They are three-dimensional. The blue elements denote that the nodes $\left(5,:,:\right)$ construct a triangle. **(e)** The corresponding adjacency tensor $A^{\left(2\right)}$ is a three-dimensional structure. The cyan-colored elements indicate that the nodes i,j, and k form a triangle.

Therefore, the values of η relate to the case of mixed positive and negative interactions. Meanwhile, the adjacency matrix of second-order coupling is denoted by $A^{\left(2\right)}=\left[a_{ijk}^{\left(2\right)}\right]$ , which shows that nodes *i,j,* and *k* can construct a triangle. Similarly, $a_{ijk}^{\left(2\right)}=1$ if nodes i,j and k have a positive second-order interconnection, and $a_{ijk}^{\left(2\right)}=-1$ if nodes i,j and k have a negative second-order interconnection. Moreover, $a_{ijk}^{\left(2\right)}=0$ means that nodes *i,j,* and *k* cannot construct a triangle. The value of $a_{ijk}^{\left(2\right)}$ is the same as the first-order coupling matrix. Furthermore, we assume that the network is undirected and unweighted. Furthermore, we assume that the nodes are connected with the global coupling patterns. Without loss of generality, to describe the topological structure of a complex network with various interactions, a five-node complex network is shown in Figure 2. As an illustration, a link (first-order) and a triangle (second-order) are depicted in Figure 2, respectively.

As we know, the Kuramoto-like model (Rodrigues et al., 2016; Acebrón et al., 2005) is regarded as a standard reduced model that characterizes the collective phenomena, which is of great interest in power systems. Before proceeding, we represent the power system as a network of generators and consumers connected by transmission lines. The starting point of our analysis is coupled second-order oscillators:

$$\frac{2H_{i}}{\omega_{R}}{\ddot{\theta }}_{i}+\frac{D_{i}}{\omega_{R}}{\dot{\theta }}_{i}=P_{i}+K_{ij}\sum_{j=1,j\neq i}^{N}\sin\left(\theta_{j}-\theta_{i}\right)$$ (2)

for i=1,⋯,N , where N denotes the number of nodes, and $\theta_{i}$ represents the phase angle of oscillator *i.* The parameters $H_{i},D_{i}$ denote inertia and damping constants, respectively. $\omega_{R}$ is the reference frequency of the system. The parameter $P_{i}$ is related to the power of node *i:* $P_{i}$ is positive for generators, while it is negative for the consumer. $K_{ij}$ represents the coupling strength between nodes.

In what follows, we focus on a higher-order coupled power system with mixed coupling patterns. First, the synchronous stability of the power system is studied for first- and second-order coupling strengths. Here, we focus on the first-order interaction of the oscillator is diffusive, that is $h_{1}\left(X_{i},X_{j}\right)=\left[\sin\left(\theta_{i}-\theta_{j}\right),0\right]$ , and for the second-order communication, we consider diffusive coupling $h_{2}\left(X_{i},X_{j},X_{k}\right)=\left[\sin\left(\theta_{j}+\theta_{k}-2\theta_{i}\right),0\right]$ .

For simplicity, we neglect Ohmic effects and assume that the oscillators have the same dissipative coefficients and the same moments of inertia. Under these approximations, we rewrite Equation 2 in the form of a dynamical system of first-order ordinary differential equations (ODEs) and consider a second-order connection:

$$\left\{\begin{array}{l} {\dot{\theta }}_{i}=\omega_{i}\\ {\dot{\omega }}_{i}=-\alpha \omega_{i}+P_{i}+K_{1}\sum_{j=1}^{N}a_{ij}^{\left(1\right)}\sin\left(\theta_{j}-\theta_{i}\right)+K_{2}\sum_{j=1}^{N}\sum_{k=1}^{N}a_{ijk}^{\left(2\right)}\sin\left(\theta_{j}+\theta_{k}-2\theta_{i}\right) \end{array}\right.$$ (3)

To measure the synchronization transition of the power system, the following complex order parameter is introduced (see Equation 4):

$$r\left(t\right)e^{i\psi \left(t\right)}=\frac{1}{N}\sum_{j=1}^{N}e^{i\theta_{j}\left(t\right)}$$ (4)

which is considered the average sum of the unit vectors associated with the phases of each oscillator in the complex plane. Here, the modulus of the resulting complex number is the order parameter, given by Equation 5

$$R\left(t\right)=\left|\frac{1}{N}\sum_{j=1}^{N}e^{i\theta_{j}\left(t\right)}\right|$$ (5)

In this way, $R\left(t\right)=0$ stands for the network in low synchrony, and $r\left(t\right)=1$ corresponds to high levels of synchrony.

## 3 Stability analysis of synchronous solutions

In this section, we aim to derive the synchronous solution of a power system with high-order interactions. The corresponding synchronized solution is $\omega_{s}=\omega_{1}=\cdots \omega_{N}.$ First, we define two new variables: $\chi_{i}=K_{1}\sum_{j=1}^{N}a_{ij}^{\left(1\right)}\sin\left(\theta_{j}-\theta_{i}\right),\gamma_{i}=K_{2}\sum_{j=1}^{N}\sum_{k=1}^{N}a_{ijk}^{\left(2\right)}\sin\left(\theta_{j}+\theta_{k}-2\theta_{i}\right)$ .

According to Equation 3, one can obtain Equation 6

$${\dot{\omega }}_{s}=-\alpha \omega_{s}+P_{i}+\chi_{i}+\gamma_{i}$$ (6)

It is found that variables ${\dot{\omega }}_{s},\omega_{s}$ do not relate to index *i.* Then, let

$${\dot{\omega }}_{s}+\alpha \omega_{s}=P_{1}+\chi_{1}+\gamma_{1}=\cdots =P_{n}+\chi_{n}+\gamma_{n}=\Omega_{0}$$ (7)

So Equation 7 becomes

$${\dot{\omega }}_{s}=\Omega_{0}-\alpha \omega_{s}$$ (8)

Suppose that ${\dot{\omega }}_{s}=0$ , the power system achieves frequency synchronization. Hence, Equation 8 becomes the form of Equation 9.

$$\omega_{s}=\frac{1}{\alpha }\Omega_{0}$$ (9)

Afterwards, the value of the parameter $\Omega_{0}$ can be calculated by Equation 7

$$\sum_{i=1}^{N}\left[-\alpha \omega_{s}+P_{i}+K_{1}\sum_{j=1}^{N}a_{ij}^{\left(1\right)}\sin\left(\theta_{j}-\theta_{i}\right)+K_{2}\sum_{j=1}^{N}\sum_{k=1}^{N}a_{ijk}^{\left(2\right)}\sin\left(\theta_{j}+\theta_{k}-2\theta_{i}\right)\right]=0$$ (10)

Because the power system is symmetric, we can then obtain Equation 11.

$$K_{1}\sum_{j=1}^{N}a_{ij}^{\left(1\right)}\sin\left(\theta_{j}-\theta_{i}\right)+K_{2}\sum_{j=1}^{N}\sum_{k=1}^{N}a_{ijk}^{\left(2\right)}\sin\left(\theta_{j}+\theta_{k}-2\theta_{i}\right)=0$$ (11)

Subsequently, Equation 10 can be rewritten as Equation 12.

$$\sum_{i=1}^{N}\left[-\alpha \omega_{s}+P_{i}\right]=0\Rightarrow \omega_{s}=\frac{1}{N\alpha }\sum_{i=1}^{N}P_{i}=\frac{1}{\alpha }\bar{P}$$ (12)

where $\bar{P}$ denotes the average value of all $P_{i}$ . Without loss of generality, we assume that $P_{i}^{Generator}>0$ at the generators, and $P_{i}^{Consumer}<0$ at the consumers, and the total consumption equals the total amount of generation, that is, $\sum_{i=1}^{N}P_{i}=0$ . Thus, the average value of $\omega_{s}=0.$

In the following, to assess the linear stability of the synchronization, the linear stability analysis is developed on a general network model with the first- and second-order interactions by considering a small perturbation to the synchronous manifold $X_{s}$ as $X_{i}=\delta X_{i}+X_{s}$ . First, a new variable $\delta X={\left[\delta X_{1}^{T},\delta X_{2}^{T},\cdots ,\delta X_{N}^{T}\right]}^{T}$ is defined. Then, we have that

$$\begin{array}{l} \dot{\delta }X_{i}=Jf\left(X_{s}\right)\delta X_{i}+K_{1}\sum_{j=1}^{N}a_{ij}^{\left(1\right)}\times \left[\frac{\partial h_{1}\left(X_{i},X_{j}\right)}{\partial X_{i}}{|}_{\left(X_{s},X_{s}\right)}\delta X_{i}+\frac{\partial h_{1}\left(X_{i},X_{j}\right)}{\partial X_{j}}{|}_{\left(X_{s},X_{s}\right)}\delta X_{j}\right]\\ \begin{array}{cc} & \begin{array}{l} +K_{2}\sum_{j=1}^{N}\sum_{k=1}^{N}a_{ijk}^{\left(2\right)}\times [\frac{\partial h_{2}\left(X_{i},X_{j},X_{k}\right)}{\partial X_{i}}{|}_{\left(X_{s},X_{s},X_{s}\right)}\delta X_{i}+\frac{\partial h_{2}\left(X_{i},X_{j},X_{k}\right)}{\partial X_{j}}{|}_{\left(X_{s},X_{s},X_{s}\right)}\delta X_{j}\\ \left.+\frac{\partial h_{2}\left(X_{i},X_{j},X_{k}\right)}{\partial X_{k}}{|}_{\left(X_{s},X_{s},X_{s}\right)}\delta X_{k}\right] \end{array} \end{array} \end{array}$$ (13)

where $Jf\left(X_{s}\right)$ represents the Jacobian matrix of the function f , assessed at the synchronous state $X_{s}$ . Here, we focus on the fact that all the coupling functions are synchronization noninvasive. In other words, their value is constant at the synchronous manifold. Hence, we get

$$\begin{array}{l} \frac{\partial h_{1}\left(X_{i},X_{j}\right)}{\partial X_{i}}{|}_{\left(X_{s},X_{s}\right)}+\frac{\partial h_{1}\left(X_{i},X_{j}\right)}{\partial X_{j}}{|}_{\left(X_{s},X_{s}\right)}=0,\\ \frac{\partial h_{2}\left(X_{i},X_{j},X_{k}\right)}{\partial X_{i}}{|}_{\left(X_{s},X_{s},X_{s}\right)}+\frac{\partial h_{2}\left(X_{i},X_{j},X_{k}\right)}{\partial X_{j}}{|}_{\left(X_{s},X_{s},X_{s}\right)}\\ +\frac{\partial h_{2}\left(X_{i},X_{j},X_{k}\right)}{\partial X_{k}}{|}_{\left(X_{s},X_{s},X_{s}\right)}=0 \end{array}$$ (14)

Moreover, we have that $\sum_{j=1}^{N}a_{ij}^{\left(1\right)}=k_{i}^{\left(1\right)},\sum_{j=1}^{N}\sum_{k=1}^{N}a_{ijk}^{\left(2\right)}=2k_{i}^{\left(2\right)}$ , based on Equation 14, the previous Equation 13 reads

$$\begin{array}{l} \left.\dot{\delta }X_{i}=Jf\left(X_{s}\right)\delta X_{i}-K_{1}\sum_{j=1}^{N}L_{ij}^{\left(1\right)}\times \frac{\partial h_{1}\left(X_{i},X_{j}\right)}{\partial X_{j}}{|}_{\left(X_{s},X_{s}\right)}\delta X_{j}\right]\\ \begin{array}{cc} & -K_{2}\sum_{j=1}^{N}\sum_{k=1}^{N}\tau_{ijk}\left[\frac{\partial h_{2}\left(X_{i},X_{j},X_{k}\right)}{\partial X_{j}}{|}_{\left(X_{s},X_{s},X_{s}\right)}\delta X_{j}+\frac{\partial h_{2}\left(X_{i},X_{j},X_{k}\right)}{\partial X_{k}}{|}_{\left(X_{s},X_{s},X_{s}\right)}\delta X_{k}\right] \end{array} \end{array}$$ (15)

Here, a tensor T is defined whose elements are $\tau_{ijk}=2k_{i}^{\left(2\right)}\delta_{ijk}-a_{ijk}^{\left(2\right)}$ . In addition, the new notations are introduced as follows (Equation 16).

$$\begin{array}{l} Jh_{1}\left(X_{s},X_{s}\right)=\frac{\partial h_{1}\left(X_{i},X_{j}\right)}{\partial X_{j}}{|}_{\left(X_{s},X_{s}\right)},\\ J^{\left(1\right)}h_{2}\left(X_{s},X_{s},X_{s}\right)=\frac{\partial h_{2}\left(X_{i},X_{j},X_{k}\right)}{\partial X_{j}}{|}_{\left(X_{s},X_{s},X_{s}\right)},\\ J^{\left(2\right)}h_{2}\left(X_{s},X_{s},X_{s}\right)=\frac{\partial h_{2}\left(X_{i},X_{j},X_{k}\right)}{\partial X_{k}}{|}_{\left(X_{s},X_{s},X_{s}\right)} \end{array}$$ (16)

According to the above definition, Equation 15 can be rewritten as

$$\begin{array}{l} \dot{\delta }X_{i}=Jf\left(X_{s}\right)\delta X_{i}-K_{1}\sum_{j=1}^{N}L_{ij}^{\left(1\right)}Jh_{1}\left(X_{s},X_{s}\right)\delta X_{j}\\ \begin{array}{cc} & \left.-K_{2}\sum_{j=1}^{N}[J^{\left(1\right)}h_{2}\left(X_{s},X_{s},X_{s}\right)\delta X_{j}\sum_{k=1}^{N}\tau_{ijk}+J^{\left(2\right)}h_{2}\left(X_{s},X_{s},X_{s}\right)\delta X_{k}\sum_{j=1}^{N}\tau_{ijk}\right] \end{array}. \end{array}$$ (17)

Based on the symmetric of the tensor *T*, Equation 17 becomes Equation 18

$$\begin{array}{l} \dot{\delta }X_{i}=Jf\left(X_{s}\right)\delta X_{i}-K_{1}\sum_{j=1}^{N}L_{ij}^{\left(1\right)}Jh_{1}\left(X_{s},X_{s}\right)\delta X_{j}\\ \begin{array}{cc} & -K_{2}\sum_{j=1}^{N}\left[J^{\left(1\right)}h_{2}\left(X_{s},X_{s},X_{s}\right)\delta X_{j}L_{ij}^{\left(2\right)}+J^{\left(2\right)}h_{2}\left(X_{s},X_{s},X_{s}\right)\delta X_{k}L_{ij}^{\left(2\right)}\right] \end{array}\\ \begin{array}{cc} & = \end{array}Jf\left(X_{s}\right)\delta X_{i}-K_{1}\sum_{j=1}^{N}L_{ij}^{\left(1\right)}Jh_{1}\left(X_{s},X_{s}\right)\delta X_{j}\\ \begin{array}{cc} & -K_{2}\sum_{j=1}^{N}L_{ij}^{\left(2\right)}\left[J^{\left(1\right)}h_{2}\left(X_{s},X_{s},X_{s}\right)+J^{\left(2\right)}h_{2}\left(X_{s},X_{s},X_{s}\right)\right] \end{array}\delta X_{j}. \end{array}$$ (18)

where $L^{\left(1\right)}=\left[L_{ij}^{\left(1\right)}\right]=K-A^{\left(1\right)}$ is the classical Laplacian matrix, which is defined as shown in Equation 19

$$L_{ij}^{\left(1\right)}=\left\{\begin{array}{l} -K_{ij},i\neq j\\ -\sum_{i\neq l}^{n}L_{il},i=j \end{array}\right.$$ (19)

Matrix *K* is the diagonal matrix with the degree of the nodes, and $A^{\left(1\right)}$ is the first-order adjacency matrix. The second-order Laplacian matrix $L^{\left(2\right)}$ is defined as shown in Equation 20

$$L_{ij}^{\left(2\right)}=\left\{\begin{array}{l} i\neq j\begin{array}{cc} : & \left\{\begin{array}{l} a_{ij}^{\left(1\right)}=0:0,\\ a_{ij}^{\left(2\right)}=1:-k_{ij}^{\left(2\right)}, \end{array}\right. \end{array}\\ i=j\begin{array}{cc} : & 2k_{i}^{\left(2\right)}\; \end{array} \end{array}\right.$$ (20)

$k_{i}^{\left(2\right)}=\frac{\left(N-1\right)\left(N-2\right)}{2}k_{ij}^{\left(2\right)}$ where is the number of triangles that contain node *i*, representing the degree of transmission link *ij*, that is, the total number of triangles having the link *ij*. Then, a tensor $T={\left[\tau_{ijk}\right]}_{N\times N\times N}$ is defined as $T=K^{\left(2\right)}-A^{\left(2\right)}$ , where the elements of $K^{\left(2\right)}=\left[k_{ijk}\right]=2k_{i}^{\left(2\right)}$ for i=j=k ; otherwise, $k_{ijk}=0$ . Moreover, we have that $\sum_{j=1}^{N}a_{ij}^{\left(1\right)}=k_{i}^{\left(1\right)},\sum_{j=1}^{N}\sum_{k=1}^{N}a_{ijk}^{\left(2\right)}=2k_{i}^{\left(2\right)}$ .

Let us rewrite Equation 18 in block form by introducing the stack vector $\delta X={\left[\delta X_{1}^{T},\delta X_{2}^{T},\cdots ,\delta X_{N}^{T}\right]}^{T}.$ Furthermore, $JF=Jf\left(X_{s}\right),JG^{\left(1\right)}=Jg^{\left(1\right)}\left(X_{s},X_{s}\right),JG^{\left(2\right)}=J_{1}g^{\left(2\right)}\left(X_{s},X_{s},X_{s}\right)+J_{1}g^{\left(2\right)}\left(X_{s},X_{s},X_{s}\right)$ , one obtains Equation 21

$$\overset{\cdot }{\delta X}=\left[I_{N}⊗JF-\sigma_{1}L^{\left(1\right)}⊗JG^{\left(1\right)}-\sigma_{2}L^{\left(2\right)}⊗JG^{\left(2\right)}\right]\delta X.$$ (21)

We assume that the eigenvectors of the classic Laplacian matrix $L_{ij}^{\left(1\right)}$ are represented by $\beta_{1},\beta_{2},...,\beta_{N}$ . Therefore, one defines new variables $\zeta =\left(B^{-1}⊗I_{m}\right)\delta X$ , where $B=\left[\beta_{1},\beta_{2},...,\beta_{N}\right].$ Then, we obtain that Equation 22

$$\dot{\zeta }=\left(B^{-1}⊗I_{m}\right)\left[I_{N}⊗JF-k_{1}L_{ij}^{\left(1\right)}⊗JG^{\left(1\right)}-k_{2}L_{ij}^{\left(2\right)}⊗JG^{\left(2\right)}\right]\left(B⊗I_{m}\right)\zeta ,$$ (22)

$I_{N}$ represents the N×N unit matrix. It is noted that generalized Laplacians are symmetric real-valued zero-row-sum matrices. Furthermore, they are all diagonalizable and the smallest eigenvalues $\lambda_{1}=0$ .Thereby, $BL_{ij}^{\left(1\right)}B^{-1}=diag\left(\lambda_{1},\lambda_{2},...,\lambda_{N}\right)=\Gamma$ , here $0=\lambda_{1}\leq \lambda_{2}\leq ...,\lambda_{N}$ are the eigenvalues of $L_{ij}^{\left(1\right)}$ . Simultaneously, $BL_{ij}^{\left(2\right)}B^{-1}=\Omega$ describe the transformed second-order Laplacian matrix. One can get

$$\dot{\zeta }=\left[I_{N}⊗JF-K_{1}\Gamma ⊗JG^{\left(1\right)}-K_{2}\Omega ⊗JG^{\left(2\right)}\right]\zeta ,$$ (23)

The following equations can be derived:

$$\left\{\begin{array}{l} {\dot{\zeta }}_{1}=JF\zeta_{1}\\ {\dot{\zeta }}_{2}=\left(JF-K_{1}\lambda_{2}JG^{\left(1\right)}\right)\zeta_{1}-K_{2}\sum_{j=2}^{N}\Omega JG^{\left(2\right)}\zeta_{2} \end{array}\right.$$ (24)

where $JF=Jf\left(X_{s}\right),JG^{\left(1\right)}=Jg^{\left(1\right)}\left(X_{s},X_{s}\right),JG^{\left(2\right)}=J_{1}g^{\left(2\right)}\left(X_{s},X_{s},X_{s}\right)+J_{1}g^{\left(2\right)}\left(X_{s},X_{s},X_{s}\right)$ denote the Jacobian matrices for the functions $f,g^{\left(1\right)},g^{\left(2\right)}$ .

Thus, the stability of the coupled oscillators can be reduced by solving Equation (24) and calculate the maximum transverse Lyapunov exponents. We note that the necessary condition for the stability of a synchronous solution requires that the maximum transverse Lyapunov exponents be negative.

## 4 Numerical simulations

In this section, numerical simulations are performed under different types of interactions coexisting within the same system to better understand the dynamics that emerge in the higher-order power system above. Here, we take Figure 1 as an example. In this work, we address the scenario of a network with first-order and second-order interactions. In each of the following cases, the maximum Lyapunov exponents of the linearized equation for N=5 are shown according to both coupling strengths. The integration is performed using the fourth-order Runge–Kutta algorithm and with a time step of $h=10^{-3}$ . In particular, the interactions include positive and negative couplings between nodes that coexist in the same network. Following this direction, we focus on the following cases to investigate the emergence of synchronized states.

### 4.1 Purely positive interconnections

For the sake of illustration, we start BY considering the case of purely positive coupling, that is,η=0 . The maximum Lyapunov exponent of the system is depicted in Figure 2a according to both coupling strengths. In general, synchronization is achieved in a smaller coupling strength $K_{1}$ , considering the second-order interactions. This threshold is decreased with an increase in the strength of the second-order interactions. The evolution curves for different second-order coupling strengths $K_{2}$ are shown in Figure 2b according to the first-order coupling strength $K_{1}$ .

**FIGURE 3:**
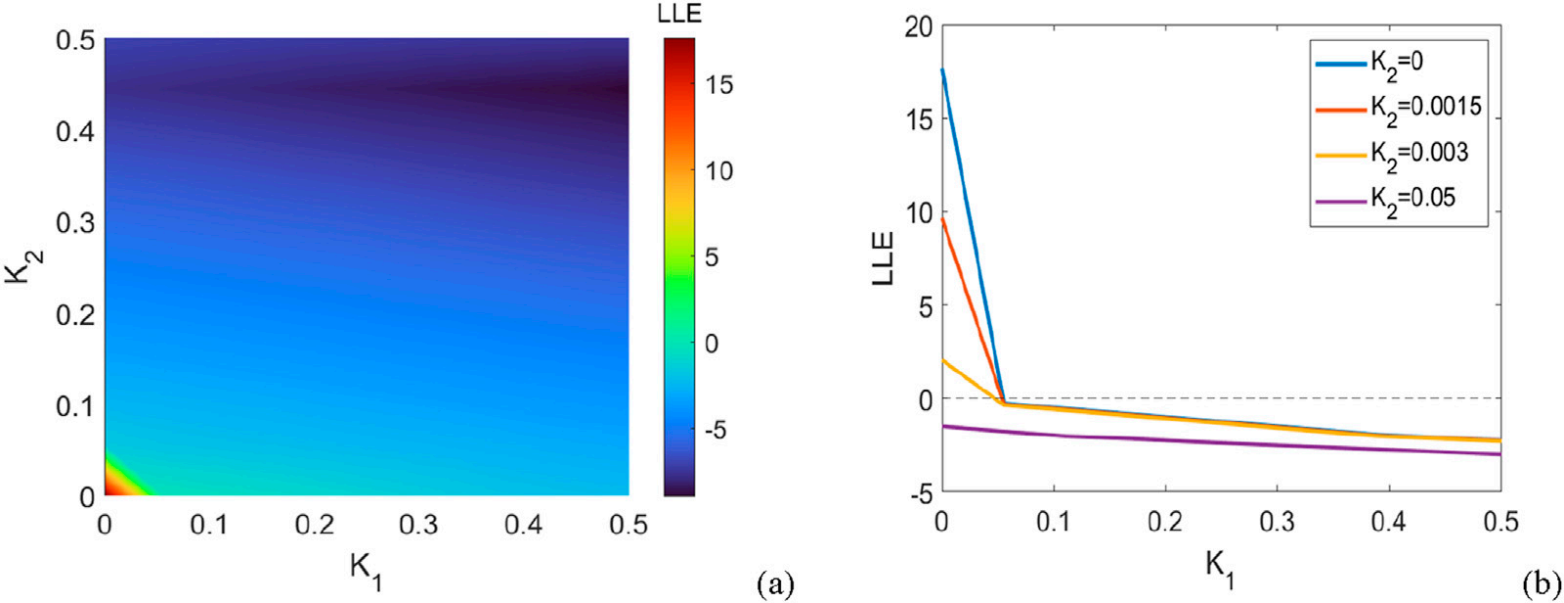
System parameters: number of oscillators, showing the regions of synchronous and asynchronous states for coupled oscillators with purely positive couplings. **(a)** The maximum Lyapunov exponent of the linearized Equation 23 in the parameter plane $\left(K_{1},K_{2}\right)$ . This plot shows the transition regions between the synchronous and asynchronous states of the coupled oscillators. The Largest Lyapunov exponent (LLE) is color-mapped, displaying synchronous regions (low LLE) and asynchronous regions (high LLE). **(b)** The effect of $K_{1}$ on the LLE for different $K_{2}$ = 0, 0.00 15, 0.003, 0.05. This plot shows how the synchronization and desynchronization behaviors evolve as $K_{1}$ changes, with different curves representing the LLE for each fixed $K_{2}$ .

According to Figure 3, we find that with increasing $K_{2}$ , a smaller first-order coupling strength $K_{1}$ ensures the synchronization. In addition, one can observe that the second-order interaction has little effect on the synchronization capability of the network within a certain range for the first-order coupling strength. For instance, for $K_{2}=0.0015$ and $K_{2}=0.003$ , the synchrony can be obtained for the same value $K_{1}=0.072$ . In addition, it is noted that the power system can achieve synchronization for any value of $K_{1}$ when $K_{2}=0.05$ .

### 4.2 Purely negative interconnections

**FIGURE 4:**
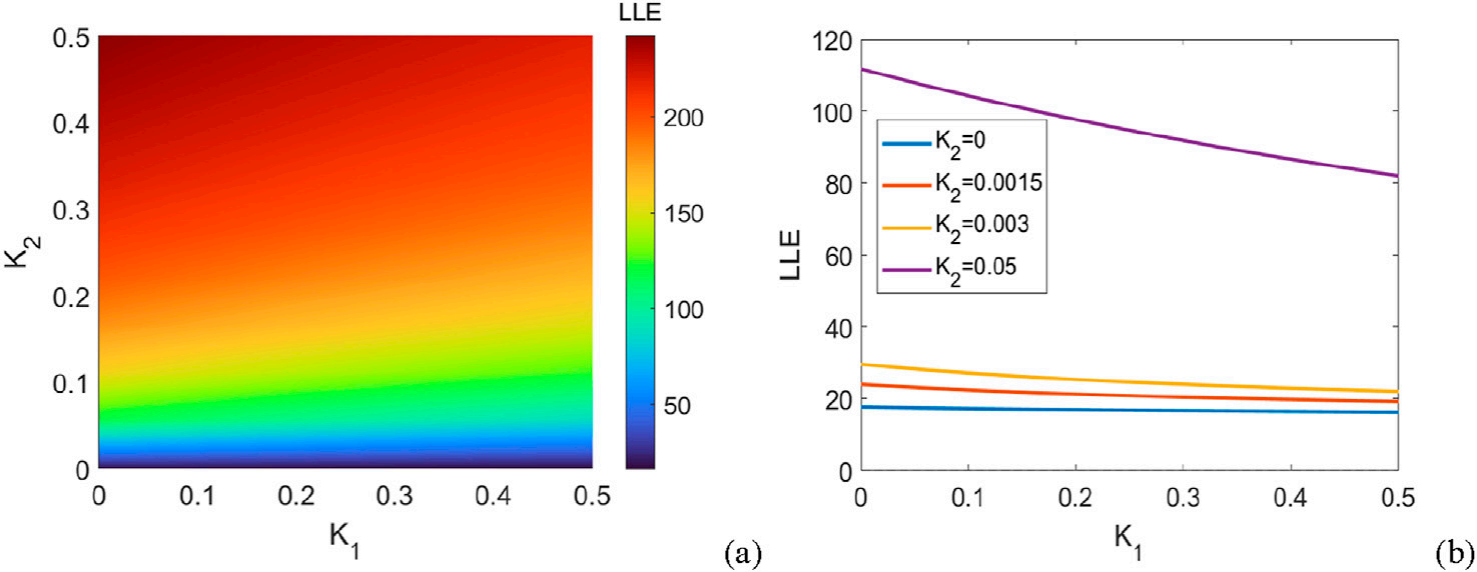
The system consists of $\left(N=5\right)$ coupled oscillators with purely negative couplings, and the figure shows the regions of synchronous and asynchronous states in the parameter plane $\left(K_{1},K_{2}\right)$ , where $K_{1}$ and $K_{2}$ are the coupling strengths between the oscillators. **(a)** The maximum Lyapunov exponent of the linearized Equation 23 in the parameter plane $\left(K_{1},K_{2}\right)$ . The plot shows the transition between stable (synchronous) and unstable (asynchronous) states of the coupled oscillators, with the LLE value color-mapped. **(b)** The description of the system behavior according to different values of $K_{2}$ (set to $K_{2}$ = 0, 0.00 15, 0.003, 0.05). The plot shows how the maximum Lyapunov exponent (LLE) changes with $K_{1}$ for each fixed value of $K_{2}$ .Each line corresponds to a different coupling value $K_{2}$ .

Then, we restrict our attention to the case of purely negative couplings, that is,η=1 . Figure 4 shows the regions of synchronization and asynchronization and the maximum Lyapunov exponent of Equation 23 for N=5 .

From Figure 4, first, it can be observed that in the absence of second-order interactions ( $K_{2}=0$ ), the oscillatory power system is unable to achieve synchronization. In addition, we find that a network with purely negative coupling still exhibits asynchronous states regardless of the varying values of second-order interactions. Hence, by combining insights from Figures 3, 4, these observations indicate that positive couplings lead to the enhancement of synchronous ability while negative couplings make the oscillators repulsive and have disadvantageous effects on synchronous ability.

**FIGURE 5:**
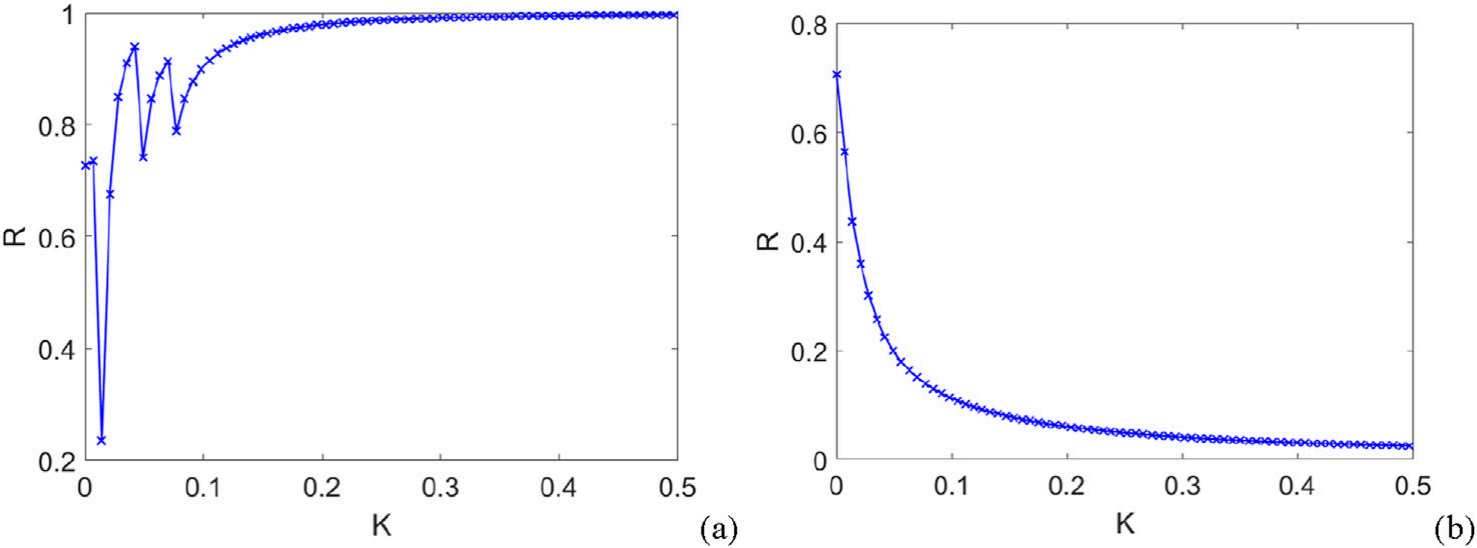
The evolution curves of the order parameter of a high-order oscillatory network with different coupling patterns. **(a)** purely positive coupling; **(b)** purely negative coupling.

Figure 5 also shows the evolution of the order parameter for the entire network under different coupling types. Here, for simplicity, we assume the coupling strengths of the first-, second-, and higher-order couplings are equal; thus, the parameter K represents a uniform coupling strength across all interactions. Based on Figure 5a, it can be observed that the order parameter approaches 1 as the first-order coupling strength increases. However, from Figure 4B, it is found that the order parameter declines with the increase in first-order coupling strength. Therefore, synchronization cannot be achieved for the negative coupling strength. This indicates that the results are comparable to a situation in which second-order coupling is disregarded. In the following section, we turn our focus to the case of mixed positive and negative couplings, that is,0<η<1 .

### 4.3 Mixed positive and negative couplings

**FIGURE 6:**
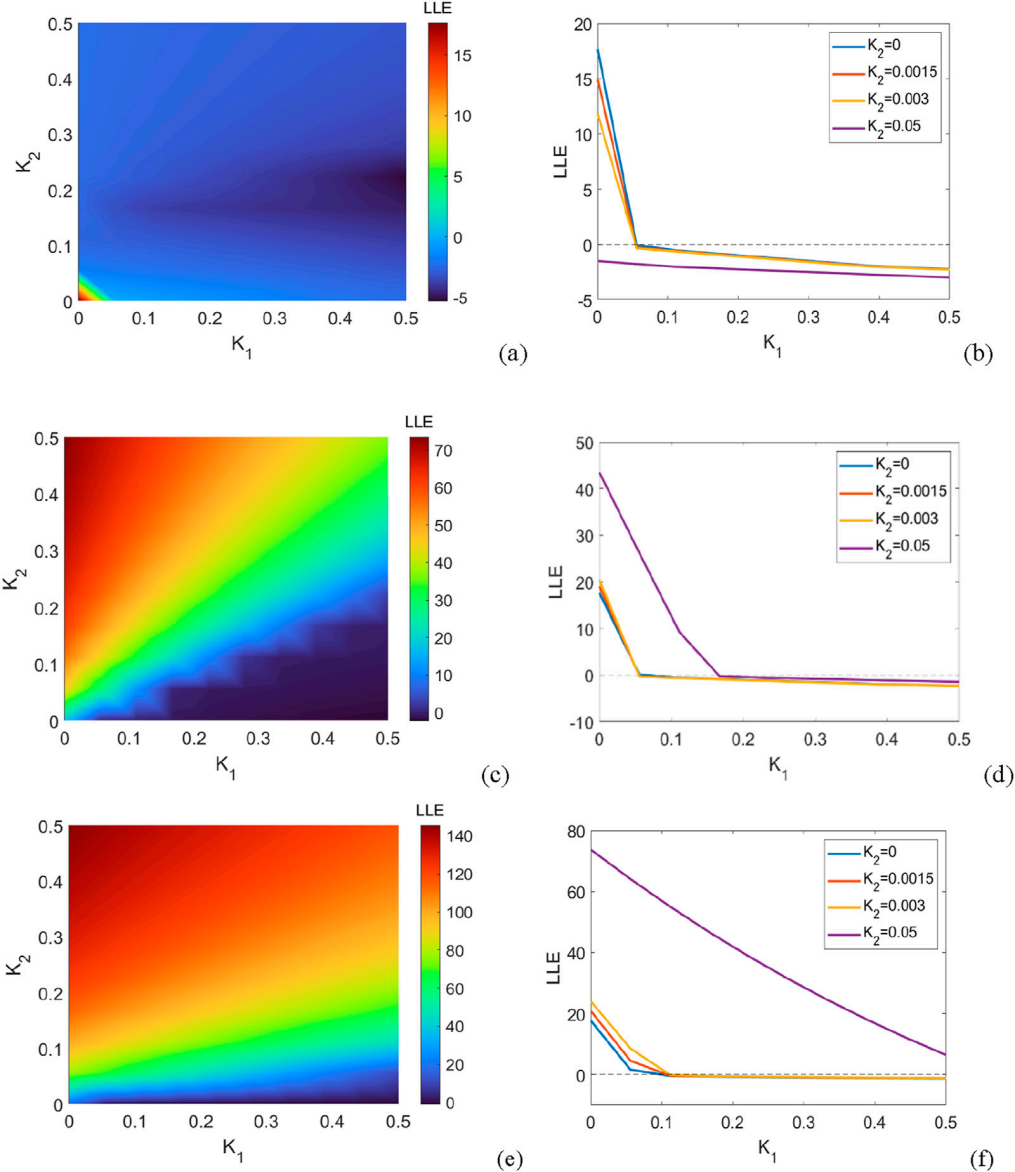
The regions of synchronous and asynchronous states for coupled oscillators with mixed negative and positive couplings. **(a,b)** η=0.2 , **(c,d)** η=0.6 , **(e,f)** η=0.8 . Where **(a,c,e)** denote the maximum Lyapunov exponent of the linearized Equation (23) in the parameter plane $\left(K_{1},K_{2}\right)$ . The blue part is the fully synchronized region of the higher-order oscillatory power system. **(b,d,f)** show the description according to $K_{1}$ for $K_{2}$ = 0, 0.00015, 0.0003, 0.05.

Here, we consider cases in which interactions can be either positive or negative. Figures 6a–f show the regions of synchronous and asynchronous states for coupled oscillators with mixed positive and negative couplings. It can be found that for a small η ,for example, η=0.2 , the synchronous region is rather large. As η increases to η=0.6 , the synchronous region becomes smaller. With further increase of η , for example, η=0.8 , the synchronous region almost disappears.

Based on the numerical results presented in Figure 6, when second-order interactions are taken into account, purely positive couplings lead to a synchronous state, while purely negative couplings can impede synchronization. Additionally, both the coupling strength and the ratio of the two types of couplings jointly shape the collective behaviors of the oscillatory network. It is important to note that the above-mentioned phenomena are derived from the global coupling of all oscillators. In other words, these numerical results are in line with those of conventional networks with either purely positive or purely negative couplings. Consequently, in small-scale oscillatory power systems, the impact of second-order interactions on the synchronous state is restricted.

The results presented above allow us to reach several conclusions about how different coupling patterns affect the synchronous stability of higher-order coupled oscillatory networks. In particular, we note that these networks are restricted to hybrid coupling types, which include binary interactions and higher-order interactions among the units. Finally, we explore the relationship between synchronizability and the coupling patterns in globally coupled networks. Here, we assume that all the couplings are positive. In what follows, simulation results are presented to illustrate the impact of first- and second-order interactions on synchronous performance.

**FIGURE 7:**
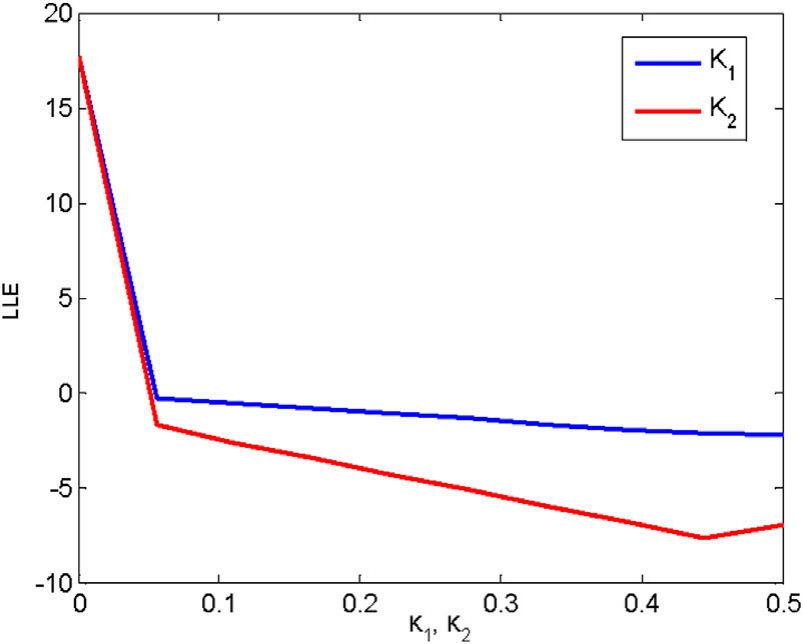
The maximum Lyapunov exponent of all-to-all coupled oscillators under different interaction patterns.

A smaller value of the maximum Lyapunov exponent implies better synchronizability; that is, a smaller coupling strength is required to realize synchronization. Here, we examine a network of 10 nodes consisting of all-to-all coupled oscillators. We estimate the synchronizability by measuring the maximum Lyapunov exponent, taking into account both pairwise and second-order interactions. Figure 7 displays the differences in the stability of the coupled network under different coupling patterns.

Figure 7 shows that a coupled network with fully second-order interactions exhibits better synchronizability than the case of first-order interactions. That is, second-order interactions enhance synchronization with respect to first-order interactions.

## 5 Conclusion

High-order networks are a powerful framework for characterizing real-world complex systems. Taking into account second-order interactions, we explore the synchronous performance of a globally higher-order network and derive the synchronous solutions of the model. In contrast to previous oscillatory models where nodes typically had uniform coupling patterns, in our model, the coupled oscillators interact according to a common pattern, which can be either positive or negative. According to the presented model, we analyze the impact of higher-order interactions on synchronous performance under different circumstances. Our findings disclose that the synchronization capability of a network with purely positive couplings is not influenced by the second-order interactions. However, the network with purely negative coupling exhibits asynchronous states for any different values of second-order interactions. In addition, with the increase of the negative coupling, the region of synchrony gradually diminishes. In summary, the coupling pattern plays a crucial role in shaping collective dynamics within globally higher-order networks. Especially for physiological networks, collective behaviors are crucial for maintaining vital life functions such as heartbeat, respiration, and cognition. The research not only reveals the principles of the robustness of living systems but also provides theoretical support for the treatment of diseases and the design of bionic systems.

Consequently, the proposed network model, along with the synchronous analysis, offers valuable perspectives for the design of more resilient real-world systems. As future work, we intend to integrate real-world data with the topological structures of networks featuring higher-order interactions to explore complex dynamic behaviors.

## Data Availability

The original contributions presented in the study are included in the article/Supplementary Material; further inquiries can be directed to the corresponding author.
