# Supplementary material for: Collective behavior of higher-order globally coupled oscillatory networks in response to positive and negative couplings
Source: Front Netw Physiol. 2025 Jul 22;5:1582297. doi: 10.3389/fnetp.2025.1582297 (PMC12321779; doi:10.3389/fnetp.2025.1582297)
Supplement: Supplementary file 1 [file Supplementaryfile1.pdf]

Supplementary Material

Collective behavior of higher-order globally coupled oscillatory network in response to positive and negative couplings

Lixin Yang[1], Mengjiao Li[1], Jun Jiang[2, *]

[1]School of Mathematics & Data Science, Shaanxi University of Science and Technology, Xi'an, 710021, China

[2] State Key Laboratory for Strength and Vibration, Xi'an Jiaotong University, Xi'an, 710049, China

*** Correspondence:**
Corresponding Author
Jun Jiang (jun.jiang@xjtu.edu.cn)

Appendix

In our numerical simulations, we used paradigmatic Kuramoto oscillators for the study of synchronization in systems of coupled units. Here, we derive the necessary condition of stability for power system according the dynamics equation. It is important to notice that, frequency-synchronized synchronous state is achieved in power system to ensure the energy transmission. Furthermore, in the case of synchronous power system, $\dot{\theta}_1 = \dot{\theta}_2 = \cdots \omega_s$, and $\omega_s = 0$ for a large scale power system. In the next step, the linear stability analysis is done on the network equation (3). Firstly, according the model of coupled oscillatory system ($X = [\theta, \omega]$), one obtain

$$\begin{cases} \delta\dot{\theta}_i = \delta\omega_i, \\ \delta\dot{\omega}_i = -\alpha\delta\omega_i - K_1 \sum_{j=1}^{N} a_{ij}^{(1)} \cos(\theta_j - \theta_i)(\delta\theta_j - \delta\theta_i) - K_2 \sum_{j=1}^{N}\sum_{k=1}^{N} a_{ijk}^{(2)} [\cos(\theta_j + \theta_k - 2\theta_i)(\delta\theta_j + \delta\theta_k - 2\delta\theta_i)]. \end{cases} \tag{25}$$

where $L^{(1)} = [L_{ij}^{(1)}] = K - A^{(1)}$ is defined as

$$L_{ij}^{(1)} = \begin{cases} -K_{ij} \cos\left(\phi_i - \phi_j\right), i \neq j \\ -\sum_{i \neq l}^{n} L_{il}, i = j \end{cases} \tag{26}$$

Matrix K is the diagonal matrix with the degree of the nodes and $A^{(1)}$ is the first-order adjacency matrix.

And the second-order Laplacian matrix $L^{(2)}$ is defined as

$$L_{ij}^{(2)}=\begin{cases} i\neq j: & \begin{cases} a_{ij}^{(1)}=0:0, \\ a_{ij}^{(2)}=1:-k_{ij}^{(2)}, \end{cases} \\ i=j: & 2k_i^{(2)}, \end{cases} \tag{27}$$

where $k_i^{(2)}=\dfrac{(N-1)(N-2)}{2}$ is the number of triangles which contains node *i*, $k_{ij}^{(2)}$ represents the degree of transmission link *ij*, i.e., the total number of the triangles having the link *ij*. Then, a tensor $T=[\tau_{ijk}]_{N\times N\times N}$ is defined as $T=K^{(2)}-A^{(2)}$, where the elements of $K^{(2)}=[k_{ijk}]=2k_i^{(2)}$ for $i=j=k$, otherwise, $k_{ijk}=0$. According to the above definition , we can get

$$\begin{cases} \delta\dot{\theta}_i=\delta\omega_i, \\ \delta\dot{\omega}_i=Jf(X_s)\delta X_i-K_1\sum_{j=1}^{N}L_{ij}^{(1)}\cos(\theta_j-\theta_i)\delta\theta_j \\ \quad -K_2\sum_{j=1}^{N}\sum_{k=1}^{N}[k_{ijk}-\tau_{ijk}]\cos(\theta_j+\theta_k-2\theta_i)[\delta\theta_j+\delta\theta_k-2\delta\theta_i] \\ \quad =Jf(X_s)\delta X_i-K_1\sum_{j=1}^{N}L_{ij}^{(1)}\cos(\theta_j-\theta_i)\delta\theta_j-K_2\sum_{j=1}^{N}\sum_{k=1}^{N}\tau_{ijk}\cos(\theta_j+\theta_k-2\theta_i)[\delta\theta_j+\delta\theta_k] \\ \quad =Jf(X_s)\delta X_i-K_1\sum_{j=1}^{N}L_{ij}^{(1)}\cos(\theta_j-\theta_i)\delta\theta_j-2K_2\sum_{j=1}^{N}L_{ij}^{(2)}[\cos(\theta_j+\theta_k-2\theta_i)]\delta\theta_j. \end{cases} \tag{28}$$

Let us rewrite (28) in block form by introducing the stack vector $\delta X=[\delta X_1^T,\delta X_2^T,\cdots,\delta X_N^T]^T$, furthermore, $JF=Jf(X_s),JG^{(1)}=Jg^{(1)}(X_s,X_s),JG^{(2)}=J_1g^{(2)}(X_s,X_s,X_s)+J_1g^{(2)}(X_s,X_s,X_s)$

For coupled oscillators system, the 1-order and 2-order Laplacians matrices can be described as follows

$$L_{ij}^{(1)}=\begin{bmatrix} -\sum_j a_{1j}^{(1)}\cos(\phi_j-\phi_1) & \cdots & \sum_j a_{1N}^{(1)}\cos(\phi_1-\phi_N) \\ \cdots & \cdots & \cdots \\ \sum_j a_{N1}^{(1)}\cos(\phi_N-\phi_1) & \cdots & -\sum_j a_{Nj}^{(k)}\cos(\phi_j-\phi_N) \end{bmatrix}, \tag{29}$$

$$L_{ij}^{(2)}=\begin{bmatrix} -\frac{(N-1)(N-2)}{2}\sum_l b_{kl}\cos(2\phi_j-\phi_{j_1}-\phi_i) & \cdots & \frac{(N-1)(N-2)}{2}\sum_l b_{kl}\cos(\phi_1^{0(k)}-\phi_N^{0(M)}) \\ \cdots & \cdots & \cdots \\ \frac{(N-1)(N-2)}{2}\sum_l b_{kl}\cos(\phi_N^{0(M)}-\phi_1^{0(1)}) & \cdots & -\frac{(N-1)(N-2)}{2}\sum_l b_{kl}\cos(\phi_j^{0(M)}-\phi_i^{0(k)}) \end{bmatrix}. \tag{30}$$

We assume that the eigenvectors of the classic Laplacian matrix $L_{ij}^{(1)}$ are represented by $\beta_1,\beta_2,...,\beta_N$.

Therefore, one define new variables $\zeta=(B^{-1}\otimes I_m)\delta X$, where $B=[\beta_1,\beta_2,...,\beta_N]$. Hence, according to Eqs.(22)-(24), the stability conditions of the coupled oscillators are obtained.
